# Emergency Department Utilization among Underserved African American Older Adults in South Los Angeles

**DOI:** 10.3390/ijerph16071175

**Published:** 2019-04-02

**Authors:** Mohsen Bazargan, James L. Smith, Sharon Cobb, Lisa Barkley, Cheryl Wisseh, Emma Ngula, Ricky J. Thomas, Shervin Assari

**Affiliations:** 1 Department of Family Medicine, Charles R. Drew University of Medicine and Science (CDU), Los Angeles, CA 90059, USA; mobazarg@cdrewu.edu (M.B.); sharoncobb@cdrewu.edu (S.C.); lisabarkley@cdrewu.edu (L.B.); 2 Department of Family Medicine, University of California, Los Angeles (UCLA), Los Angeles, CA 90095, USA; jamessmith@cdrewu.edu; 3 School of Nursing, Charles R. Drew University of Medicine and Science, Los Angeles, CA 90095, USA; 4 Department of Pharmacy Practice, West Coast University, Los Angeles, CA 90004, USA; cWisseh@westcoastuniversity.edu; 5 Department of public health, Charles R. Drew University of Medicine and Science, Los Angeles, CA 90095, USA; emmangula@cdrewu.edu; 6 Department of Emergency Medicine, UC Davis Medical Center, University of California, Davis, Sacramento, CA 95817, USA; rthomasmd18@gmail.com

**Keywords:** emergency department utilization, African Americans, older adults

## Abstract

*Objectives:* Using the Andersen’s Behavioral Model of Health Services Use, we explored social, behavioral, and health factors that are associated with emergency department (ED) utilization among underserved African American (AA) older adults in one of the most economically disadvantaged urban areas in South Los Angeles, California. *Methods:* This cross-sectional study recruited a convenience sample of 609 non-institutionalized AA older adults (age ≥ 65 years) from South Los Angeles, California. Participants were interviewed for demographic factors, self-rated health, chronic medication conditions (CMCs), pain, depressive symptoms, access to care, and continuity of care. Outcomes included 1 or 2+ ED visits in the last 12 months. Polynomial regression was used for data analysis. *Results:* Almost 41% of participants were treated at an ED during the last 12 months. In all, 27% of participants attended an ED once and 14% two or more times. Half of those with 6+ chronic conditions reported being treated at an ED once; one quarter at least twice. Factors that predicted no ED visit were male gender (OR = 0.50, 95% CI = 0.29–0.85), higher continuity of medical care (OR = 1.55, 95% CI = 1.04–2.31), individuals with two CMCs or less (OR = 2.61 (1.03–6.59), second tertile of pain severity (OR = 2.80, 95% CI = 1.36–5.73). Factors that predicted only one ED visit were male gender (OR = 0.45, 95% CI = 0.25–0.82), higher continuity of medical care (OR = 1.39, 95% CI = 1.01–2.15) and second tertile of pain severity (OR = 2.42, 95% CI = 1.13–5.19). *Conclusions:* This study documented that a lack of continuity of care for individuals with multiple chronic conditions leads to a higher rate of ED presentations. The results are significant given that ED visits may contribute to health disparities among AA older adults. Future research should examine whether case management decreases ED utilization among underserved AA older adults with multiple chronic conditions and/or severe pain. To explore the generalizability of these findings, the study should be repeated in other settings.

## 1. Introduction

As a result of the growing aging population, there has been an increase in the prevalence of chronic medical conditions (CMCs). In lieu of system-level difficulties in managing the chronic conditions of this population, the emergency department (ED) has taken on a significant role in providing care for this population [1]. Older adults account for 14.4% of the 20 million ED visits that occur each year in the US [2]. Based on the current rate of growth in healthcare utilization, by 2050 it is expected that the US health system would not be able to meet the demands of the older population who would need ED [3]. Therefore, there is a need for additional information that can potentially reduce unnecessary ED utilization in populations with CMCs.

Emergency Departments deliver important healthcare services and commonly serve as the point of entry to the hospital system or as a means of connecting patients to after-hours care, particularly those without a primary care provider (PCP). ED utilization can also be a pathway to long-term care settings. While EDs will always play a vital role, ED over-utilization remains a concern and could be reduced through better downstream management of chronic conditions. Therefore, further investigation of the health status of older adults who utilize the ED is warranted and may yield insight into the factors that contribute to ED utilization and hospitalization [4]. 

The delivery of care to the growing aging population is a pressing challenge facing the United States health care system [1]. Technological innovations, healthcare provider incentives, higher inpatient costs, and managed care policies have contributed to the shift in decreasing inpatient hospital admissions, leading to increased ED visits. Older adults with multiple comorbidities, cognitive and functional impairments, and polypharmacy may present to the ED with symptoms and signs of an acute physical and/or mental illness, requiring rapid triage and diagnosis [5,6,7,8]. Providing high quality and safe care for underserved older adults with multiple and complex CMCs remains challenging for ED providers. Older adults often experience adverse events after an ED visit [9]. Low neighborhood income and high comorbidity status are contributing risk factors for in-patient hospitalization within 30 days of an ED visit among older adults [10]. 

ED visits are more common for older adults, minorities, and uninsured individuals [11]. The Nationwide Inpatient Sample (NIS), a large, nationally-representative dataset of hospital discharges, shows that the odds of unscheduled ED-to-hospital admission is 39% higher for African Americans (AAs) compared to their white counterparts [11]. The ED is responsible for four out of five unscheduled hospitalizations. Furthermore, in the last decade, unscheduled hospitalization in the United States has grown in complexity, with more individuals presenting in the ED with a variety of acute clinical conditions. Traditional approaches to assessing healthcare delivery have focused mainly on primary care services, delivery of care through patient-centered homes, managed care, and accountable care organizations [12], but the issue of ED utilization has gone relatively unexamined.

The healthcare system is overwhelmed with the challenges of providing care for older adults, specifically AAs who suffer from poorer health outcomes. The 2014 National Hospital Ambulatory Medical Care Survey (NHAMCS) determined that AA older adults had higher ED visit rates than their white counterparts, including higher rates of non-urgent visits, thus indicating a trend of ED over-utilization by this population [2]. Multiple studies have established that AAs have higher ED visit rates than other ethnicities [13,14]. Interestingly, AAs who have a usual source of care or commercial health maintenance organization (HMO) coverage are more likely to use the ED compared to other people [15]. In addition, compared to whites, AAs are more likely to select the ED as their usual place for healthcare [16,17]. These disparities have been attributed to a number of factors; however, the underlying reasons remain poorly understood [18]. One study found that low income AAs preferred the ED over ambulatory care because it was more accessible, less expensive, and provided a higher quality of care [19]. Disparities within the ED exist for AAs who are less likely to receive non-invasive cardiac diagnostic tests for chest pain [20], adequate pain management [21], post-ED follow-up care for psychiatric and behavioral disorders including substance use [22], as well as cancer care [23].

### 1.1. Theoretical Framework

This study uses the Andersen’s Behavioral Model of Health Services Use [24] to examine ED visits among older AAs. The model has been used extensively to examine the use of health services by various populations, including minority groups [25,26,27,28,29,30,31]. The aim of this model is to discover potential determinants that either facilitate or impede the utilization of health services among various populations by observing the interactions of relevant features of health care, including individual, social, and contextual factors [32]. Specifically, the model examines the inequities of healthcare access and has the potential to inform health-service policy and delivery. Initially developed in the 1960s, the model has undergone five phases of transformation, with a specific focus on contextual and individual determinants. The major components of both contextual and individual determinants are, first, predisposing characteristics; second, enabling factors; and, third, perceived and evaluated need as potential predictors for health service utilization. The fourth component is health-seeking behavior, which is comprised of individual health practices including health service use [24,33].

Predisposing factors encompass demographic and socio-cultural characteristics, including personal health beliefs, social structure, and political perspectives [34]. Enabling factors include decision-making factors to use a healthcare resource, incorporating community and organizational values, and system/structural elements [35]. Need factors are subdivided into perceived need and evaluated need [36]. Perceived need refers to the patient’s own sense of need for health services, including self-judged severity of health and illness symptoms. Evaluated need refers to objective measures of the patient’s medical and healthcare needs as determined by healthcare providers or other professionals [25]. Both health and functional status can be measured within the perceived need and evaluated need constructs. Incidence and progression of CMCs are both impacted by the quantity and quality of health service utilization [37]. Comprehensive, multilevel assessment of individual and contextual differences in health-services utilization on the part of older AAs remains an urgent need.

### 1.2. Aims

Using Andersen’s Behavioral Model of Health Services Use [24], we conducted this study to explore social, behavioral, and health factors that are associated with ED utilization on the part of underserved populations. We were particularly interested in populations with multiple risk factors such as older age, economic disadvantage, and minority racial status. As a result, we studied determinants of ED use among underserved AA older adults in one of the most economically disadvantaged urban areas in south Los Angeles, California--Service Planning Area 6 (SPA6).

## 2. Materials and Methods

### 2.1. Design and Setting

This cross-sectional study included a convenience sample of 609 non-institutionalized underserved AA older adults, aged 65 years and older. Participants were recruited from 11 senior housing units, 16 predominantly AA churches, and one public housing project located in SPA6 in Los Angeles County.

### 2.2. Participants and Samling 

Participants were eligible if they were AA, 65 years or older, and were able to complete an interview in English. Participation in any other clinical trials, being institutionalized in a health care setting, and considerable cognitive impairment were exclusion criteria.

### 2.3. Comparability of Our Sample

Our participants were comparable to AA older adults in South LA. About 35% of our sample had a high school diploma. Similar to our sample, in the California Health Interview Survey (CHIS) data, 37% of AAs aged 65 years or older in South Los Angeles had a high school diploma [38]. Regarding the health status of the participants, one third of the participants described their self-rated health (SRH) as fair (29%) or poor (5%). Similar to our data, CHIS data has shown that 38% of AA older adults (65 years or older) living in South Los Angeles report their SRH as fair or poor [38]. 

### 2.4. Measurements

The study employed face-to-face structured interviews. The survey toolkit was a collection of several validated measures from various sources [26,39,40,41,42,43].

#### 2.4.1. Enabling Factors

*Financial Difficulty*. Financial difficulty was measured using a five-item measure with items that were on a five-point Likert scale (1 = always to 5 = never) [44]. Participants were asked in the last 12 months how frequently they were unable to: (1) buy the amount of food their family should have; (2) buy the clothes they feel their family should have; (3) pay their rent/mortgage; (4) pay their monthly bills; and (5) make ends meet. A higher score was indicative of less financial difficulty within the last 12 months (α = 0.934).

*Continuity of Medical Care.* Continuity of medical care was measured using three items. Participants were asked to report (1) what type of place they usually visit to receive medical care (a private doctor’s office/private medical group vs other settings); (2) whether they usually go to the same place for medical care; and (3) whether they are usually seen by the same health provider when they receive medical care. The answer to the second and third items were yes = 1, no = 0, and the response to the first question was coded private office = 1 versus any other place = 0. The total score ranged from 0–3, where a higher score indicated more continuity of care.

*Accessibility of Medical Care.* Accessibility of medical care was measured using three items. Participants were asked how difficult it is for them to (1) “visit a doctor when you need medical care”; (2) “get a routine physical examination if you wanted one”; and (3) “travel to medical appointments”. A high score indicates less difficulty.

*Satisfaction with Medical Care.* Satisfaction with Medical Care was measured using three items. They were asked “Overall, how satisfied are you with (1) the medical care you currently receive; (2) how available medical care is for you; and (3) your access to preventative services (i.e., routine checkups). A high score indicates less satisfaction.

*Demographic Factors.* Age (interval variable) and gender (dichotomous variable) were the demographic factors. 

#### 2.4.2. Perceived and Evaluated Need for Care Characteristics

*Intensity of Pain*. Pain intensity was captured using the Short-Form McGill Pain Questionnaire 2 (SF-MPQ-2) [41]. In structured interviews, participants reported the extent to which they experienced each type of pain. Overall, 22 pain items measured experience of bodily pain in the past week. All items were on an 11-point scale (0 = “none” to 10 = “worst possible”). The SF-MPQ-2 provides a total pain score that is an average of all questions [41]. A high score is indicative of greater pain intensity (α = 0.945). We categorized level of pain based on the tertile of the variable (first, second, and third tertiles).

*Depressive Symptoms.* We used the Geriatric Depression Scale (Short Form) (GDS-SF) to measure severity and frequency of depressive symptoms. This measure uses 15 items that are on a “yes”/ “no” response scale [43]. The scale provides a total score which varies between 0 and 15, where a higher score indicates more depressive symptomatology. The GDS-SF has excellent reliability and validity. It is widely used to measure depressive symptoms in older adults in the community, as well as acute and long-term care settings [45].

*Self-Rated Health (SRH)*. This study measured SRH by the following single question: “In general, would you say your health is (1) Excellent; (2) Very good; (3) Good; (4) Fair; and (5) Poor?” This single item has been frequently used in health research, national surveys, and longitudinal cohorts [42].

*Chronic Medical Conditions (CMCs)*. Number of CMCs was measured by asking whether participants have been diagnosed with the following conditions: (1) asthma or bronchitis; (2) arthritis; (3) high blood pressure; (4) heart problems; (5) diabetes mellitus; (6) back pain/injury; (7) depression; (8) cancer; (9) thyroid problems; (10) sleeping/insomnia; (11) stroke; (12) migraine headache; and (13) stomach or intestinal problems.

#### 2.4.3. Outcome

*Emergency Department (ED) Visits.* Participants were asked how many times they had utilized ED in the last 12 months. Responses were coded as 0, 1, or 2+.

### 2.5. Statistical Analysis

Univariate, bivariate, and multivariable statistical methods were applied using the Statistical Package for Social Sciences (SPSS) version 22 (SPSS Inc., Chicago, IL, USA). We reported frequencies and means for univariate analysis. At bivariate level, chi-square test, independent samples t-test, and analysis of variance (ANOVA) were employed to explore correlation between ED service utilizations and (1) predisposing; (2) enabling; and (3) need-for-care characteristics. In addition, multinomial logistic regressions were applied to test the association between independent variables and ED utilization. We used the “Enter” rather than the “Forward” or “Backward” method. Thus, independent variables were kept in the model even if they were not statistically significant. Our independent variables were selected based on an extensive literature review informed by our theoretical framework. Andersen’s Behavioral Model of Health Services Use mainly focuses on: (1) predisposing; (2) enabling; and (3) need-for-care characteristics.

### 2.6. Ethics

The current investigation was approved by the Institutional Review Board (IRB) at the Charles R. Drew University of Medicine and Science (CDU; IRB #14-12-2450-05). Written informed consent was received from all the participants. Data were kept confidential at all stages of research.

## 3. Results

### 3.1. Sample

Table 1 provides a descriptive view of the sample. The current analysis included 609 AA individuals who were 65 years or older (mean, 74 ± 7). About 35% of the participants were male. About 16% of the sample was currently married or lived with a partner/significant other.

Table 2 shows the CMCs in our sample. The overall number of CMCs ranged between 1 and 10 (mean: 4.2 ± 2.1). Thirty-four percent of participants reported being diagnosed with diabetes mellitus and 58% reported having chronic back pain. Almost one out of four participants reported that they suffer from asthma or chronic bronchitis. Three out of four older AAs received care from two or more physicians. In addition, 68% reported that they accessed medical care at a private doctor’s office or private medical group.

### 3.2. Emergency Department Visits

About 41% of our participants were treated at an ED during the last 12 months prior to the interviews. Approximately 27% and 14% of participants were treated once and at least twice in the ED during the last 12 months, respectively (Table 1). More than 19% of men and 11% of women were treated at least twice in the last 12 months in the ED. In addition, 50% and 25% with 6+ CMC received care at the ED once or at least twice within last 12 months, respectively (Table 1).

### 3.3. Bivariate Correlates of ED Utilization

Table 1 and Table 2 report bivariate correlations between independent variables and ED utilization. Table 1 indicates that gender, continuity and accessibility of medical care, number of CMCs, SRH, intensity of pain, and depressive symptoms all are significantly associated with ED utilization. Among various CMCs, asthma, heart disease, depression, stroke, migraine headache, and GI-related problems were positively associated with ED utilization (Table 2).

### 3.4. Multivariate Correlates of ED Utilization

Table 3 shows the results of multinomial logistic regression models on the effects of (1) predisposing; (2) enabling; and (3) need-for-care characteristics on ED utilization. The table shows odds ratios (ORs) and 95% confidence intervals (CIs) for each independent variable. Table 3 indicates that gender is the only predisposing variable that is associated with ED utilization. Compared to women, men had 2.0 (1/0.50 = 2) and 2.2 (1/0.045 = 2.2) times less odds of “no ED” or “one ED visit” as opposed to two or more ED visits during the last 12 months. Controlling for predisposing variables, the enabling factor of “continuity of care” was found to be a significant correlate of ED utilization. Respondents who indicated a lower level of continuity of care had 1.55 (1.04–2.31) higher odds of reporting at least two ED visits compared to no ED visits within the last 12 months.

After controlling for predisposing and enabling characteristics, the multinomial logistic regression showed two of the need-for-care variables as significant predictors of ED utilization: (1) number of CMCs and (2) severity of pain. Depressive symptoms and SRH were not significant in multivariate analysis, even though they were significant in bivariate analysis. Respondents with a higher number of CMCs (>2) had 2.61 and 1.87 higher odds of two ED and one ED visits within the last 12 months, compared to participants with one or no CMC.

Table 3 indicates that after controlling for other predisposing, enabling, and need-for-care factors, pain intensity was associated with ED visit. Respondents in first or second tertile of pain intensity had 2.8 and 1.76 lower odds of having no ED visit or only one ED visit compared to those who were in the highest tertile of pain intensity. Odds of having at least two ED visits (compared with one ED visit) increased 2.42 times among participants who were in the highest tertile of pain intensity. 

## 4. Discussion

We found that 27% and 14% of older AAs in SPA6, one of the most economically disadvantaged areas in South Los Angeles, had at least one or two ED visits within a one-year period. Previous studies have found that AAs are more likely than whites to have ED visits [14,46,47,48]. Indeed, AAs have two-times higher odds of having the ED as their usual healthcare source than their white counterparts [16]. Furthermore, frequent ED use is associated with poor quality of life [49]. Frequent ED use disrupts the continuum of care among older adults [50]. As a result, frequent ED users are at an increased risk of subsequent hospitalization, readmission, nursing home placement, and death [50]. 

In our study, the multivariate analyses introduced a range of predisposing, enabling, and need-for-care determinants of ED utilization. First, AA men were more likely to visit the ED frequently than AA women. One hypothesis is that men are less likely to engage in preventive and continuity of care than women, leading to increased visits to the ED. A considerable literature has shown the central role of gender in shaping attitudes and behaviors related to health care use. For example, a study documented that compared to AA men, AA women have lower rates of early diagnostic cardiac testing in the ED when reporting chest pain [20]. Furthermore, AA women with private insurance have fewer inpatient hospitalizations and ED visits, compared to those with public insurance [51]. This suggests that women with public insurance may have a greater financial burden and may perceive less benefit in a visit to the ED, leading to delays in accessing care. Culturally, older AA women are identified as the matriarchs of their families and usually provide care and significant resources to their immediate families and extended relatives. AA women may be burdened with so many responsibilities that their health takes a “back seat” to others in the family.

With regard to enabling factors, this study found that continuity of care was associated with fewer ED visits among this sample of older AAs. One recent study showed that patients who live in medically underserved areas (defined as areas with a lower density of primary care clinics) have a higher incidence of preventable ED utilization [52]. Other studies have documented that low versus high continuity of care is associated with greater risk of frequent ED utilization [50]. Additionally, it has been established that AAs have a longer wait time in ED when compared to whites [53,54,55]. A recent study found a 30% longer wait time for AAs as opposed to whites [56]. In addition, the ED length of stay is longer for this target population [57]. Therefore, it is reasonable to assume that lack of continuity of care, a higher rate of frequent ED visits, combined with a longer waiting time at ED are significant factors contributing to health disparities among AA older adults. Current research shows that continuity of care improves quality of care, improves patient outcomes, reduces hospitalizations and decreases ED visits, particularly among older adults with CMCs that need self-management (e.g., heart disease, diabetes, hypertension, and asthma) [58]. Improving and maintaining continuity of care among older adults have the potential to reduce health disparities. Programs that increase continuity of care should be developed and implemented among undeserved AA older adults. 

Pain severity and number of CMCs were revealed as the significant “need-for-care” variables for ED utilization. It is not surprising that higher pain severity leads to ED utilization. Overall, AAs report a higher pain level than whites when appearing in the ED [59] and a lower rate of being prescribed pain analgesics. Systematic reviews on race/ethnicity and chronic pain in the United States show that AAs are more likely to suffer untreated pain compared to whites [60]. Additionally, it is well known that minority patients are often under-evaluated and under-treated for their painful conditions in the ED [61,62]. Moreover, racial/ethnic disparities in pain management persist due to limited access to health care, inappropriate analgesic use and limited access to or under-utilization of pain specialists [63]. Empirical evidence suggest that older AAs have a higher risk for severe pain compared with whites [64]. Management of both multiple CMCs and psychological distress are strong predictors of severe pain which may explain racial/ethnic differences in pain severity [64]. In addition to an accurate diagnosis and high psychosocial functioning, pain management of AA older adults with multiple CMCs and comorbid cognitive decline benefits from a multidisciplinary approach that involves pharmacotherapy, physical rehabilitation, psychological support, and various other interventions [65]. Our findings are similar to other studies that have called for robust translational research programs on the causes and consequences of chronic pain that are specifically designed to reduce and eliminate disparities in management of care [61].

Successful case management of AA older adults with multiple CMCs requires development and implementation of well-designed care-continuum that prioritises patient safety. Delivery of health care in an interrupted, fragmented, or uncoordinated manner contributes to increased healthcare costs and inappropriate healthcare use [66]. Improving continuity of care in this context requires careful coordination across the healthcare continuum [67]. Successful communication between EDs and primary care providers in acute and community settings is an essential component of such interventions and programs [67]. A recent systematic review that evaluated the effectiveness of several types of interventions and programs (such as care plans, case management, printout case notes, diversion strategies, and social work practitioners) targeting adult frequent ED users, showed that some interventions are effective in reducing visit frequency and improving patient outcomes [68]. While not directly addressed by our study, ED case management (ED-CM) may be an appropriate intervention to decrease frequent ED utilization among underserved AA older adult patients, particularly those with multiple CMCs who suffer from pain. Case management programs have been adopted at multiple health systems as a method of improving care and decreasing costs [69]. Moreover, specialized ED case management programs are currently being implemented with a centered interdisciplinary team of physicians, nurses, social workers, and other healthcare team members. A specific focus is placed on coordination of care and communication, cross-cultural competencies, counseling, and specialized care referrals [49]. Limited available data demonstrate that frequent ED users who utilize case management programs services report a higher quality of life and improved biopsychosocial health [49]. Multiple studies also report substantial reductions in rates for ED visits and Medicare spending after implementation of a case management program [70,71,72]. One study found that individuals who accept community case management services have 55% fewer ED visits and 61% fewer hospitalizations compared to those who refused case management services [73]. Another study found that ED visits were lowered by 49% and ED length of stay decreased by 39%, attributed to the case management programs at their hospital [74]. ED-CM programs for older adults, especially AAs, must consider customized cultural and aging interventions to assist with disease management and pain treatment. Given the limited resources available in underserved areas (such as SPA6 in South Los Angeles where our sample was recruited), priority should be given to minority older adult patients with multiple CMCs with severe pain, especially those with limited access to and lack of continuity of care. 

In the bivariate analyses, we also observed a statistically significant relationship between depressive symptoms and ED utilization. However, multivariate analysis showed no association between these two variables. The association between depressive symptoms and ED utilization was subsumed by including the number of CMCs in the analysis. African American older adults with multiple CMCs may experience depressive symptoms and be at risk for major depressive disorder. Management of multiple CMCs may also lead to higher depressive symptoms [72,75,76,77,78]. A systematic review of 40 articles by Read and colleagues found that individuals with multimorbidity are twice as likely to have a depressive disorder and there is a 45% increased odds of depression with each additional CMC [79]. African American older adults have multiple CMCs, so it is imperative that health care providers screen AA older adults, especially frequent ED users, for comorbid CMCs and depression. Implementing a routine self-administered questionnaire in the ED can identify older adults at high risk of depression. 

Therefore, our health care system is facing two notable phenomena regarding ED utilization among AA older adults, both clearly pointing to major health disparities. First, AA older adults may have higher utilization of the ED compared to whites, particularly for non-urgent care. Second, AAs are at increased risk of non-scheduled hospital admissions, compared to their white counterparts. Indeed, little attention has been paid to ED utilization in AA older adults. A greater understanding of the factors that account for acute care utilization in the AA population will provide healthcare providers with strategies that can facilitate more effective utilization of ED services, promote the proper utilization of health services, improve their health status, and decrease over-crowded EDs. 

The current study was conducted in SPA6 south LA. Being home to over 1,000,000 residents, SPA6 is disproportionately affected by various health disparities relative to the rest of Los Angeles County [80]. For example, the age-adjusted diabetes death rate in SPA6 is 37.6 per 100,000 population which is almost five times higher than that in West Los Angeles (SPA5). Similarly, age-adjusted coronary heart disease death rate in the SPA6 is 147.5 per 100,000 population, compared to 87.7 in SPA5. While 32.5% of adults in SPA6 reported difficulty with accessing medical care, only 13.1% of SPA5 residents experienced these issues [80]. This evidence supports a pressing need for in-depth analyses of health care access and utilization among underserved older AAs in SPA6.

This study has implications. Providing proper and safe care for minority older adult patients with uncontrolled chronic conditions remains challenging for ED providers. There is a need for additional research to identify older adults who “frequently use ED” in the under-served and under-resourced urban community in order to design culturally appropriate interventions that can ultimately reduce unnecessary ED visits and improve the quality of care for this segment of our population.

### Limitations

Several limitations of this study should be considered before the results are interpreted and applied by clinicians, program planners, and policy-makers. First of all, the research did not collect data from participants’ medical records. We relied on self-reported data. Second, information regarding health services utilization was limited. We could collect data on nuances and details such as cause of ED use. Third, the study did not use a random sampling. We recruited a convenience sample that limits the generalizability of our results. To compare with other estimates, given the non-probability sampling methods of this study, future research may conduct comparisons by probability samples to describe the methods used to draw the sample, collect the data, and make inferences. However, our study generated comparable estimates to estimates obtained from other surveys that used probability sampling methods. This is the reason we compared our estimates of education and SRH to the estimates obtained from the CHIS survey. We did not have data on ED visits using the CMS Medicare database. Other data sets like the CMS Medicare database could provide more confidence in the accuracy of the estimates given in this study. Fourth, the study did not have any whites or any other racial and ethnic groups. As a result, we do not know if ED is higher or lower, and whether unique processes impact ED utilization in AA older adults. Fifth, some differences may exist between AA men and women regarding determinants of ED use. This study did not explore gender differences in causes and consequences of ED utilization. Finally, design of this study was cross-sectional, which limits any causal inferences. More research is needed with data collected beyond a single point in time. Nevertheless, this study is a unique community-based research that employed face-to-face interviews with a relatively large sample of AA older adults that was conducted in an underserved and under-resourced area of South Los Angeles. Still, the results should be regarded as preliminary. 

## 5. Conclusions

Despite its limitations, the results of the current study suggest avenues for future research on ED utilization patterns of low income urban older AAs with multiple CMCs. Future longitudinal research should examine the relationship between ED visit and ED-to-hospital inpatient admission. Future research may also study predisposing, enabling and need-for-care characteristics over time to understand the temporal causality between these factors. We encourage further research to explore causes and patterns of frequent ED utilization among older AAs. We also encourage a shift toward personalized patient-centered delivered systems. This research demonstrates a need to explore ways to enhance the communication between primary care and ED providers to support patients in engaging in continuity of care settings. A key factor in these patient-centered delivery systems is socially and culturally effective and responsive care that engages patients to meet their needs. Given the impact of pain and CMCs on ED utilization, improvement of disease management and control of pain may be regarded as key components in reducing ED over-utilization in lower income urban older AAs with multiple CMCs.

## Figures and Tables

**Table 1:** Participants’ characteristics by emergency department utilization within last 12 months.

Characteristics	Frequency (%) [Mean ± SD] *n* = 609	Emergency Department Visits			*p*-Value ***
		No Visit *n* = 362	One Visit *n* = 163	≥ 2 Visits *n* = 84	
***Predisposing Characteristics***					
Gender *					
Male	214 (35)	124 (58)	49 (23)	41 (19) ^a^	0.012
Female	397 (65)	238 (60)	114 (29)	43 (11) ^a^	
**Age**					
65–75	355 (58)	216 (61)	88 (25)	51 (14)	0.42
≥ 75	255 (42)	146 (58)	75 (30)	33 (13)	
**Education**					
< High school diploma	155 (25)	85 (55)	44 (28)	26 (17)	
High school diploma	214 (35)	121 (57)	57 (27)	35 (16)	
≥ Some college	242 (40)	156 (65)	62 (26)	23 (9)	
**Marital Status**					0.189
Married/living with companion	99 (16)	67 (68)	21 (21)	11 (11)	
Not married	510 (84)	295 (58)	142 (28)	73 (14)	
***Enabling Characteristics***					
**Financial Difficulty**	[4.4 ± 0.98]	[4.4 ± 0.90]	[4.4 ± 0.95]	[4.2 ± 1.2]	0.084
**Continuity of Medical Care** **	[2.6 ± 0.61]	[2.6 ± 0.60] ^a^	[2.5 ± 0.54]	[2.4 ± 0.76] ^a^	0.023
**Accessibility of Medical Care** **	[14.1 ± 1.7]	[14.2 ± 1.6] ^a^	[13.9 ± 1.8]	[13.8 ± 2.1] ^a^	0.05
**Satisfaction with Medical Care**	[7.8 ± 2.2]	[7.7 ± 2.1]	[7.7 ± 2.10]	[8.3 ± 2.5]	0.146
***Need-for-Care Characteristics***					
**Number of Chronic Conditions** *					
0–2	125 (21)	88 (70) ^a^	28 (22)	9 (8) ^b^	0.0001
3–5	341 (56)	206 (60)	94 (28)	41 (12)	
≥ 6	141 (23)	67 (48) ^a^	40 (28)	34 (24) ^b^	
**Self-Rated Health Status** *					
Fair–Poor	209 (34)	102 (49) ^a^	62 (30)	44 (21) ^c d^	0.001
Good	231 (38)	152 (66) ^b^	54 (24)	24 (10) ^c^	
Excellent–Very good	170 (28)	108 (64) ^a b^	46 (27)	16 (9) ^d^	
**Severity of Pain** **					
No or minor pain	221 (36)	149 (67) ^a^	49 (22)	23 (10) ^c^	0.0001
Moderate pain	173 (29)	108 (62) ^b^	52 (30)	13 (8) ^d^	
Severe pain	214 (35)	105 (49) ^a b^	61 (29)	48 (22) ^c d^	
**Disability Status**					
No	428 (70)	262 (61)	115 (27)	51 (12)	0.1
Yes	180 (30)	99 (55)	48 (27)	33 (18)	
**Depressive Symptoms** *	[2.6 ± 2.5]	[1.9 ± 2.2] ^a^	[2.1 ± 2.5] ^b^	[3.3 ± 3.2] ^a b^	0.0001

^a,b,c,d^ reflect pairwise significant difference. * Pearson chi square test. ** independent samples t test. *** *p*-value for comparison of all groups.

**Table 2:** Unadjusted associations between individual chronic medical conditions and emergency department utilization (*n* = 609)

Chronic Medical Conditions	Frequency (%) *n* = 609	Emergency Department Visits N (%)			*p*-Value *
		No Visit *n* = 362	One Visit *n* = 163	≥ 2 Visits *n* = 84	
Asthma or Bronchitis					
No Yes	466 (77) 141 (23)	290 (62) ^a^ 71 (50) ^a^	123 (26) 39 (28)	53 (11) ^b^ 31 (22) ^b^	0.003
Arthritis					
No Yes	206 (34) 401 (66)	126 (61) 235 (59)	55 (27) 107 (27)	25 (12) 59 (15)	0.669
High Blood Pressure					
No Yes	58 (10) 548 (90)	31 (53) 329 (60)	18 (31) 144 (26)	9 (16) 75 (14)	0.621
Heart Problems					
No Yes	425 (70) 182 (30)	263 (62) ^a^ 98 (54) ^a^	115 (27) 47 (26)	47 (11) ^b^ 37 (20) ^b^	0.009
Diabetes					
No Yes	398 (66) 209 (34)	233 (59) 128 (61)	109 (27) 53 (25)	56 (14) 28 (13)	0.809
Back Pain/Injury					
No Yes	255 (42) 352 (58)	161 (63) 200 (57)	66 (26) 96 (27)	28 (11) 56 (16)	0.158
Depression					
No Yes	518 (85) 89 (15)	322 (62) ^a^ 39 (44) ^a^	136 (26) 26 (29)	60 (12) ^b^ 24 (27) ^b^	0.0001
Cancer					
No Yes	532 (88) 75 (12)	320 (60) 41 (55)	144 (27) 18 (24)	68 (13) 16 (21)	0.133
Thyroid Problems					
No Yes	539 (89) 68 (11)	319 (59) 42 (62)	146 (27) 16 (24)	74 (14) 10 (15)	0.821
Sleeping/Insomnia					
No Yes	462 (76) 145 (24)	279 (60) 82 (56)	127 (28) 35 (24)	56 (12) 28 (19)	0.088
Stroke					
No Yes	528 (87) 79 (13)	327 (62) ^a^ 34 (43) ^a^	135 (26) ^b^ 27 (34) ^b^	66 (13) ^c^ 18 (23) ^c^	0.004
Migraine Headache					
No Yes	533 (88) 74 (12)	324 (61) ^a^ 37 (50) ^a^	142 (27) 20 (27)	67 (13) ^b^ 17 (23) ^b^	0.042
Stomach/Intestinal Problems					
No Yes	436 (72) 171 (28)	274 (63) ^a^ 87 (51) ^a^	113 (26) 49 (29)	49 (11) ^b^ 35 (21) ^b^	0.004

^a,b,c,d^ reflect pairwise significant difference, * *p*-value for comparison of all groups.

**Table 3:** Multinomial logistic regression models on determinants of ED service utilization among African American older adults using polynomial regression **.

Characteristics	No Visit	One Visit
***Predisposing Characteristics***		
**Gender**		0.45 (0.25–0.82) *
Male	0.50 (0.29–0.85) *	1
Female	1	
**Age**		0.92 (0.51–1.66)
65–75	1.18 (0.68–2.04)	1
≥ 75	1	
**Education**		0.85 (0.40–1.81)
< High school diploma	0.77 (0.38–1.54)	0.73 (0.38–1.43)
High school diploma	0.65 (0.35–1.21)	1
≥ Some college	1	
**Marital Status**		1.00 (0.43–2.28)
Married or living with companion	0.66 (0.32–1.39)	1
Not married	1	
		
***Enabling Characteristics***		0.97 (0.71–1.33)
**Financial Difficulty**	0.89 (0.67–1.18)	1.39 (1.01–2.15) *
**Continuity of medical care**	1.55 (1.04–2.31) *	0.97 (0.82–1.15)
**Accessibility of medical care**	1.07 (0.91–1.25)	0.95 (0.83–1.09)
**Satisfaction with medical care**	0.99 (0.87–1.12)	
		
***Need-for-Care Characteristics***		
**Number of Chronic Medical Conditions**		1.87 (0.68–5.13)
0–2	2.61 (1.03–6.59) *	1.51 (0.78–2.94)
3–5	1.70 (0.92–3.13)	1
≥ 6	1	
**Self-Rated Health Status**		0.62 (0.29–1.35)
Fair–Poor	0.50 (0.24–1.02)	0.83 (0.37–1.85)
Good	1.04 (0.50–2.18)	1
Excellent–Very good	1	
**Severity of Pain**		1.13 (0.53–2.40)
No or minor pain	1.76 (0.89–3.48)	2.42 (1.13–5.19) *
Moderate pain	2.80 (1.36–5.73) *	1
Severe pain	1	
**Disability Status**		0.96 (0.52–1.76)
No	0.90 (0.52–1.58)	1
Yes	1	0.92 (0.82–1.02)
**Depressive symptoms**	0.93 (0.85–1.03)	

*n* = 609; Reference: At least two emergency department admissions within last 12 months; **Nagelkerke R-Square: 14.0; −2log Likelihood = 1049.8; df = 34; Sig: 0.0001.
